# Synergistic Interactions Between Natural Phenolic Compounds and Antibiotics Against Multidrug-Resistant *K. pneumoniae*: A Pooled Analysis of 216 In Vitro Tests

**DOI:** 10.3390/microorganisms13112497

**Published:** 2025-10-30

**Authors:** Victor-Pierre Ormeneanu, Corina Andrei, Anca Zanfirescu, Ciprian Pușcașu, Octavian Tudorel Olaru, Simona Negreș

**Affiliations:** Faculty of Pharmacy, “Carol Davila” University of Medicine and Pharmacy, Traian Vuia 6, 020956 Bucharest, Romania; victor-pierre.ormeneanu@drd.umfcd.ro (V.-P.O.); ciprian.puscasu@umfcd.ro (C.P.); octavian.olaru@umfcd.ro (O.T.O.); simona.negres@umfcd.ro (S.N.)

**Keywords:** multidrug-resistant *Klebsiella pneumoniae*, phenolic compounds, antibiotic adjuvants, carbapenem resistance

## Abstract

The rapid global emergence of multidrug-resistant (MDR) *Klebsiella pneumoniae* threatens public health, as treatment options remain limited and resistance to last-line antibiotics is rising. Natural phenolic compounds emerge as promising adjuvants to restore antibiotic activity. This study pooled data from 216 in vitro assays evaluating interactions between phenolic compounds and conventional antibiotics against MDR *K. pneumoniae*. Fractional inhibitory concentration index (FICI) values were analyzed at the individual-test level, and structure–activity relationships were explored using a binary chemotype flagging approach. Overall, synergy was highly context-dependent, varying by both antibiotic class and phenolic chemotype. Polymyxin B combined with resveratrol demonstrated the most consistent and robust synergy (median FICI = 0.25, synergy rate = 96.2%), with no antagonism observed. For carbapenems, meropenem showed strong synergy when paired with flavonoids containing catechol or gallol motifs (e.g., quercetin, kaempferol), whereas curcumin exhibited inconsistent or antagonistic effects. Variability analysis revealed that combinations with low dispersion, such as polymyxin B + resveratrol, offer greater translational potential than high-variability pairs. These findings highlight the structural determinants of synergy and support further preclinical evaluation of select phenolic compounds as adjuvants to conventional antibiotics in the fight against MDR *K. pneumoniae*.

## 1. Introduction

The global rise of multidrug-resistant (MDR) *Klebsiella pneumoniae* represents a significant public health threat, particularly in clinical settings where treatment options are increasingly limited [1,2]. This Gram-negative opportunistic pathogen has developed various resistance mechanisms, including the production of extended-spectrum β-lactamases, carbapenemases, and alterations in membrane permeability, rendering it unresponsive to most available antibiotics [3]. Infections caused by MDR *K. pneumoniae* are associated with high morbidity, mortality, and substantial healthcare burdens [4,5].

Current treatment options are usually limited to last-line agents such as polymyxins (e.g., colistin), tigecycline, and aminoglycosides, frequently used in combination to enhance efficacy and reduce resistance emergence [6,7]. Novel β-lactam/β-lactamase inhibitor combinations, such as ceftazidime–avibactam and meropenem–vaborbactam, have expanded therapeutic possibilities, particularly against carbapenemase-producing strains [8]. However, resistance to these newer agents is already emerging [9,10,11,12].

Thus, the development of new antimicrobials has failed to keep pace with the rapid emergence of resistance [13], underscoring an urgent need for innovative approaches to combat MDR pathogens [14,15]. One promising avenue is the optimization of combination regimens and the use of adjuvant compounds capable of restoring or enhancing the activity of existing antibiotics [14,16]. Among these strategies, natural compounds—particularly phenolic secondary metabolites derived from plants—have attracted increasing attention due to their diverse biological activities and a relatively low potential for driving resistance [17,18,19,20]. Unlike many secondary metabolites that exert singular or indirect antimicrobial effects, phenolics possess multiple functional groups—particularly hydroxylated aromatic rings—that enable direct interaction with bacterial targets [20,21,22,23]. These structures confer membrane-disruptive capacity, efflux pump inhibition, metal chelation, enzyme modulation, and redox activity, all of which are directly relevant to reversing antimicrobial resistance and potentiating antibiotic activity [20,24,25,26,27,28,29,30,31,32,33,34,35,36,37,38,39]. Phenolic compounds additionally interfere with quorum sensing (QS), the bacterial communication system that regulates biofilm formation and virulence factor expression [40,41]. Consequently, they disrupt signal production, receptor binding, and downstream gene activation, resulting in weaker biofilms, reduced motility, and diminished toxin secretion [42,43,44,45,46,47,48,49,50,51]. Beyond bacterial targets, phenolic compounds exhibit host-protective properties, including the downregulation of pro-inflammatory cytokines, the enhancement of antioxidant defenses, and the preservation of endothelial barrier integrity [52,53,54,55,56,57,58,59].

Here in, we aim to provide an in-depth analysis of the synergistic interactions between phenolic compounds and antibiotics against MDR *K. pneumoniae* strains. This study delivers the first pooled, assay-level analysis of phenolic–antibiotic combinations, integrating reproducibility metrics and a chemotype-based structure–activity framework. By synthesizing data on minimum inhibitory concentrations (MICs), fractional inhibitory concentration indices (FICIs), and underlying molecular mechanisms, we have effectively assessed the potential of these combinations against critical priority pathogens. This approach allows clear differentiation between consistent synergistic effects and non-reproducible interactions, thereby enhancing the predictive and translational relevance of phenolic adjuvants.

## 2. Materials and Methods

A systematic search was conducted in PubMed, Web of Science, and Google Scholar using the following keywords and Boolean operators: (“phenolic” OR “polyphenol” OR “flavonoid”) AND (“antibiotic” OR “antimicrobial” OR “antibacterial”) AND (“synerg*” OR “combination” OR “FICI” OR “checkerboard” OR “fractional inhibitory concentration”) AND (“Klebsiella pneumoniae” OR “MDR Klebsiella”). The search covered all articles published up to June 2024. Only articles published in English were considered eligible for inclusion. We performed title/abstract-level screening and removed duplicate records. All remaining articles were assessed at the full-text level.

Studies were included if they reported standardized checkerboard or micro-broth dilution assays evaluating interactions between purified natural phenolic compounds and conventional antibiotics against multidrug-resistant (MDR) *K. pneumoniae* and reported quantitative data (MIC, FICI).

Studies were excluded if they met any of the following criteria:

- Use of non-standardized crude extracts or essential oils.
- Lack of reported FICI values or MIC data for both single agents and their combinations.
- Use of *K. pneumoniae* isolates susceptible to polymyxins, carbapenems, quinolones, or cephalosporins.
- Duplicate data or overlapping isolate collections.

Following screening and duplicate removal, 13 studies met the inclusion criteria, yielding a total of 216 isolate-level records for analysis [60,61,62,63,64,65,66,67,68,69,70,71,72]. Our analysis was conducted at the assay level, treating each isolate-specific test as an independent experimental unit and pooling in vitro outcomes rather than aggregating study-level summary effect sizes.

For each isolate, the following variables were recorded:

- Antibiotic name and mechanistic class;
- Natural phenolic compound;
- MIC values for each agent alone and in combination;
- FICI values.

All statistical analyses were performed in R version 4.3.2. FICI values were treated as continuous variables. When studies reported FICI values as ‘<0.5’, these were assigned a value of 0.5 using a conservative truncation approach, consistent with established methodology to avoid arbitrary imputation and prevent artificial inflation of synergy estimates [73,74].

Global synergy was evaluated using a one-sample Wilcoxon signed-rank test. Between-class differences were assessed using the Kruskal–Wallis test, followed by Dunn–Bonferroni post hoc comparisons.

To assess reproducibility, variability in FICI values was calculated for each antibiotic–compound combination using the range, interquartile range (IQR), and standard deviation (SD). Based on these metrics, combinations were classified as follows:

- Robust: median FICI ≤ 0.5 and range ≤ 0.5;
- Inconsistent: median FICI ≤ 1.0 and range > 2.0;
- Moderate: not meeting the above thresholds;
- Inconclusive: fewer than three replicate observations (n < 3).

These thresholds were empirically defined to capture both the strength and consistency of synergistic effects, following established interpretive FICI guidelines and emphasizing inter-strain variability as a determinant of translational potential.

To explore structural determinants of synergy, we curated one canonical structure per natural compound (single parent, neutral, dominant tautomer) and assigned a priori binary chemotype flags (flavonoid, stilbene, phenolic acid, simple phenol, curcuminoid/Michael acceptor, glycoside, alkaloid) and a catechol/gallol substructure flag. FICI values were analyzed at the individual-assay level; for distributional contrasts we used log_2_(FICI). Synergy and antagonism were defined as FICI ≤ 0.5 and FICI ≥ 4.0, respectively. For categorical outcomes (synergy rates by flag), Fisher’s exact test was applied and effect sizes were reported as odds ratios (OR) and risk differences (RD). Continuous contrasts of log_2_(FICI) between flagged vs. non-flagged groups were evaluated with the Mann–Whitney test, and median shifts were summarized using the Hodges–Lehmann estimator; approximate fold-changes in FICI were derived from the median difference in log_2_(FICI). All analyses were performed in R (v4.3.2).

## 3. Results

### 3.1. Synergistic Activity of Natural Phenolic Compounds with Conventional Antibiotics

**Figure 1:**
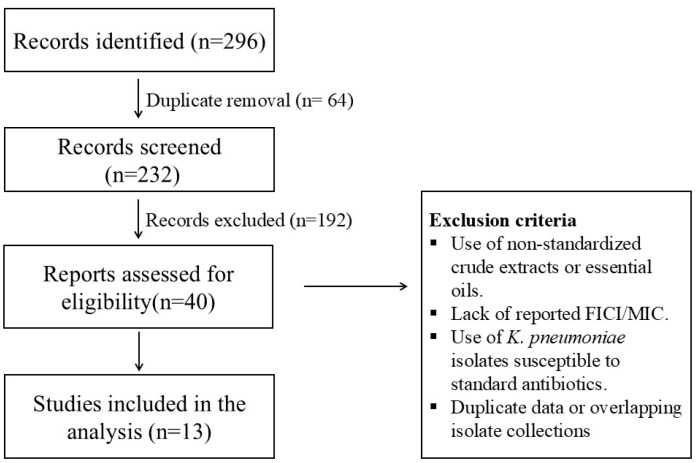
PRISMA 2020 flow diagram illustrating the study selection process for in vitro synergy assays between phenolic compounds and antibiotics against MDR Klebsiella pneumoniae.

A PRISMA 2020 flow diagram was constructed to illustrate the study selection process. The initial database search identified a total of 296 records. After the removal of duplicates, 232 unique records were screened based on title and abstract. Of these, 40 full-text articles were assessed for eligibility according to predefined inclusion and exclusion criteria, resulting in a final set of 13 studies included in the pooled analysis (Figure 1). Our dataset comprises a total of 270 in vitro synergy tests evaluating the interaction between 18 unique combinations of conventional antibiotics and natural phenolic compounds [60].

Table 1 below presents a comparative summary of the in vitro interactions between selected antibiotics and natural phenolic compounds against MDR *Klebsiella pneumoniae* strains. A FICI ≤ 0.5 indicates synergistic interaction, while a FICI between 0.5 and 1.0 is generally interpreted as additive. A FICI > 4.0 indicates an antagonistic effect [75].

Among the antibiotic–natural compound combinations tested, substantial variability in synergistic activity was observed, as reflected by the range and median FICI values. Several combinations demonstrated strong synergistic potential, suggesting their promise in restoring antibiotic efficacy against MDR *K. pneumoniae* strains.

The combination of polymyxin B and resveratrol showed the most consistent synergy, with a median FICI of 0.199 and minimal variability (range: 0.002–0.502), based on 24 independent tests. Similarly, combinations of colistin and kaempferol or eugenol exhibited a very low median FICI of 0.26 or 0.27, respectively, indicating strong synergism. However, this finding is based on a limited number of replicates (n = 6). Notably, meropenem in association with baicalein yielded the lowest FICI recorded in the dataset (0.07). However, this result was derived from a single assay and is reported solely as a hypothesis-generating signal aligned with the broader structure–activity pattern of flavonoid synergy against carbapenems. Only combinations supported by sufficient replicates and low inter-assay variability were prioritized for translational interpretation.

Conversely, combinations such as cefotaxime + matrine (median FICI = 1.050) and ciprofloxacin + myricetin (median FICI = 1.34) failed to demonstrate synergy, indicating that not all phenolic compounds contribute beneficially to antibiotic activity. Furthermore, combinations involving cinnamaldehyde with either cefotaxime or ciprofloxacin displayed high variability.

Interestingly, within the same antibiotic class, the efficacy of natural compounds varied considerably. For example, meropenem combined with quercetin demonstrated moderate synergy, while its pairing with curcumin yielded a higher median FICI.

### 3.2. Comparative In Vitro Synergistic Effects of Natural Phenolic Compounds with Key Antibiotics Against MDR K. pneumoniae

The combinations of natural phenolic compounds and antibiotics tested against MDR *K. pneumoniae*, including only those with optimal median FICI values and enough replicates to allow for robust statistical analysis, are included in Table 2.

**Figure 2:**
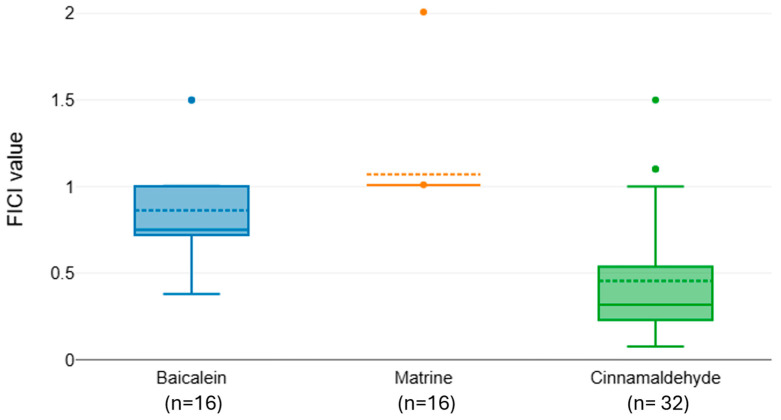
Distribution of FICI values for cefotaxime combined with baicalein, matrine, and cinnamaldehyde.

**Figure 3:**
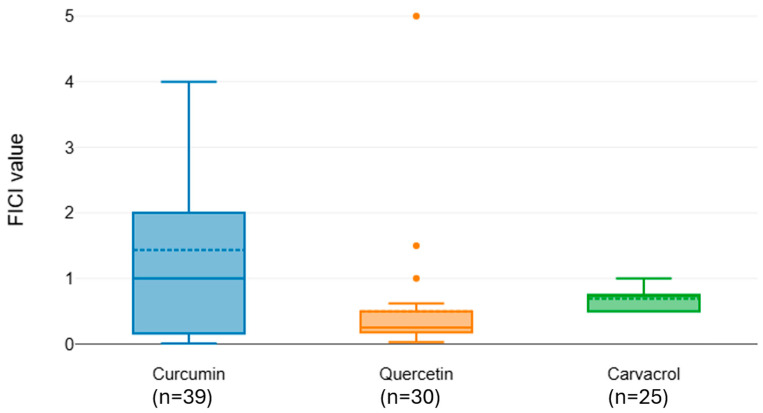
Distribution of FICI values for meropenem combined with natural phenols.

**Figure 4:**
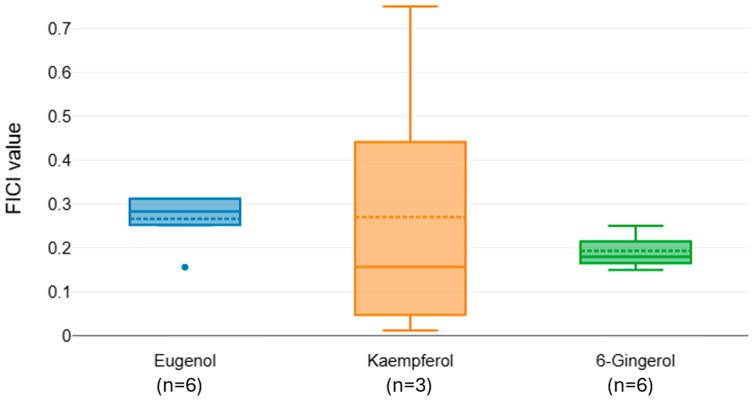
Distribution of FICI values for colistin combined with natural phenols.

Pairwise comparisons within antibiotic groups confirmed that some phenolics consistently outperform others. To better visualize pairwise differences in synergy among the natural compounds tested in combination with individual antibiotics, we generated boxplots of FICI distributions for each antibiotic group (Figure 2, Figure 3 and Figure 4). The interaction profiles of cefotaxime with the tested natural compounds revealed statistically significant differences in FICI values (Kruskal–Wallis χ^2^ = 31.77, df = 2, *p* < 0.001). Post hoc Dunn–Bonferroni analysis grouped baicalein (median FICI = 0.82) and matrine (median FICI = 1.07) in the same statistical subset (CLD = A), indicating predominantly additive to indifferent effects. In contrast, cinnamaldehyde exhibited a markedly lower median FICI (0.455) and belonged to a distinct subset (CLD = B), suggesting a synergistic interaction with cefotaxime against the tested isolates (Figure 2).

Meropenem exhibited significant variation in interaction outcomes with different natural compounds (Kruskal–Wallis χ^2^ = 16.83, df = 2, *p* < 0.001). Curcumin (median FICI = 1.436) and carvacrol (median FICI = 0.695) were classified within the same statistical group (CLD = A), indicating no consistent synergy. Quercetin, however, showed a substantially lower median FICI (0.5) and was placed in a separate group (CLD = B), consistent with a synergistic effect when combined with meropenem (Figure 3).

The FICI distributions for colistin in combination with eugenol, kaempferol, and 6-Gingerol indicate overall strong synergistic interactions, with median values below 0.5 for all compounds (Figure 4). Post hoc comparisons revealed no significant differences between pairings. Despite all compounds showing synergistic potential, 6-Gingerol yielded the lowest and most consistent FICI values, highlighting it as the most promising adjunctive agent to colistin among those tested. However, the small sample sizes (n = 3–6) limit the strength of our conclusions.

### 3.3. Synergistic Potency vs. FICI Variability

Although the synergistic potential of natural phenolic compounds combined with conventional antibiotics against MDR *K. pneumoniae* has been established in several instances, the clinical relevance of such findings requires not only high potency, but also consistent, predictable outcomes. The FICI is a robust quantitative metric for synergy assessment; however, relying solely on its median value can obscure variability across isolates.

To address this, we performed a dispersion-based analysis of FICI values for the most studied antibiotic–compound pairs (Table 3).

Analysis of dispersion metrics revealed notable differences in variability between combinations. Cefotaxime + matrine displayed exceptionally low variability, indicating highly consistent additive effects across replicates. Similarly, meropenem + carvacrol and colistin + eugenol exhibited narrow ranges (0.5 and 0.156, respectively) and low SD values (0.173 and 0.062), suggestive of stable interaction profiles. In contrast, meropenem + curcumin and meropenem + quercetin exhibited substantial dispersion, with wide ranges (3.991 and 4.97, respectively) and large SD values (1.266 and 0.900), reflecting variable interaction outcomes from strong synergy to pronounced antagonism.

Cefotaxime + baicalein and cefotaxime + cinnamaldehyde demonstrated moderate dispersion (IQR 0.28 and 0.314, respectively), with FICI values spanning both synergistic and indifferent interaction ranges. In contrast, the stability of cefotaxime + matrine suggests a reproducible interaction type with minimal fluctuation.

The observed variability in FICI values has important implications for prioritizing combinations for further development. Combinations with low dispersion are more likely to demonstrate reproducible efficacy in subsequent in vivo models, making them strong candidates for preclinical testing. High-dispersion combinations, while potentially capable of producing strong synergy, may be less reliable due to inconsistency, which could stem from strain-specific responses, compound instability, or experimental variability. Thus, dispersion-based analysis complements traditional mean FICI interpretation by identifying both robust and unstable interaction profiles, guiding rational selection of candidates for translational research.

**Figure 5:**
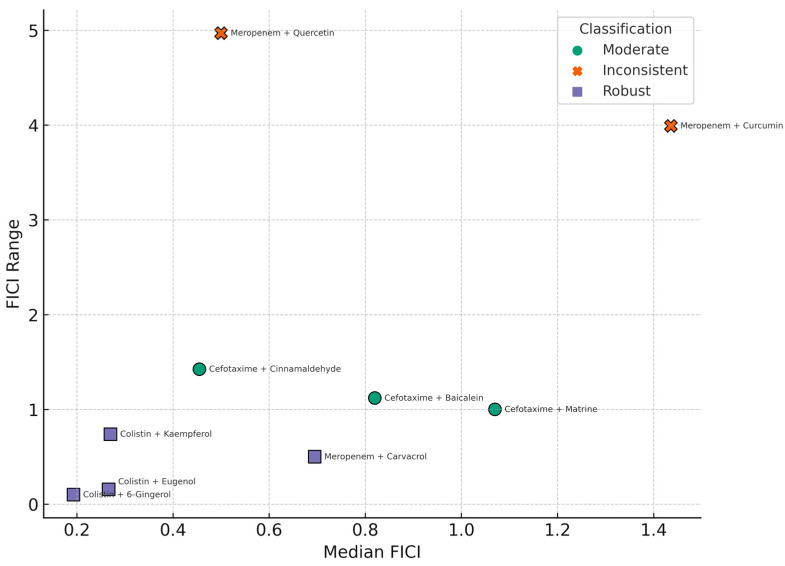
Scatterplot showing the relationship between synergistic potency (median FICI) and variability (FICI range) across 11 antibiotic–phenolic compound combinations tested against multidrug-resistant *K. pneumoniae*. Each point represents one combination and is color-coded by robustness classification: robust (median FICI ≤ 0.5 and range ≤ 0.5), inconsistent (median FICI ≤ 1.0 and range > 2.0), moderate (other qualifying combinations), and inconclusive (n < 3). Vertical and horizontal dashed lines indicate the 0.5 thresholds used for defining synergy and consistency, respectively.

The robustness classification of antibiotic–phenolic compound combinations based on synergy metrics is illustrated in Figure 5.

This analysis highlights that FICI dispersion is a critical component in evaluating the translational potential of antibiotic-phenolic combinations. Highly variable combinations, even with low median FICI values, may not consistently yield synergistic effects across diverse clinical strains. Therefore, such inconsistencies must be considered when prioritizing candidates for preclinical development or clinical trials.

### 3.4. SAR Analysis

We next asked whether simple, mechanism-motivated chemotype flags could predict functional outcomes. We curated one canonical structure per compound (single parent, neutral, dominant tautomer) and assigned binary flags a priori (flavonoid, stilbene, phenolic acid, simple phenol, curcuminoid/Michael acceptor, glycoside, alkaloid; plus a catechol/gallol substructure flag), as seen in Table 4.

FICI values were analyzed at the individual-test level. For distributional contrasts we used log_2_(FICI). Table 5 summarizes the distribution of synergy and antagonism across structural classes of natural products, as well as continuous contrasts of log_2_(FICI) values. These analyses identify structural motifs associated with enhanced or reduced combinatorial activity.

The analysis demonstrated distinct structure–activity patterns among the tested natural products. Flavonoids showed a significantly higher synergy rate (64.9%) compared to non-flavonoids (23.8%), with an odds ratio of 5.94 and a 2.7-fold reduction in median FICI, indicating a robust and reproducible potentiating effect. This trend was even more pronounced for compounds containing catechol or gallol substructures, which exhibited the highest synergy rate among all groups (80.9%) and a fourfold reduction in FICI. Conversely, curcuminoids and other Michael acceptor motifs showed lower synergy rates (30.8%) relative to compounds lacking this feature (44.9%), with a slight increase in median FICI, indicating a trend toward neutral or antagonistic interactions.

Simple phenols demonstrated reduced synergy (28%) and a statistically significant negative risk difference (−28.8%), suggesting that small, monofunctional phenolic scaffolds lack sufficient structural complexity to potentiate antibiotics effectively in this context.

Phenolic acids displayed a nominal synergy rate of 100%; however, this was based on a limited sample size (wide confidence interval) and should be interpreted cautiously. Alkaloids showed no synergistic activity (0%), with a strongly significant negative association, indicating that this chemotype is unsuitable for antibiotic potentiation against *K. pneumoniae*.

## 4. Discussion

The primary objective of this study was to systematically evaluate the potential of natural phenolic compounds to enhance the activity of conventional antibiotics against MDR *Klebsiella pneumoniae*, a critical priority pathogen. By pooling and analyzing 216 in vitro assays, we aimed to identify combinations with consistent synergistic interactions, explore variability in synergy across different antibiotic–compound pairs, and provide a SAR perspective to guide the rational selection of adjunctive agents. This approach addresses an urgent clinical need to restore the efficacy of existing antibiotics amid rapidly increasing resistance and a lag in the slow pace of new antibiotics development [76,77].

Phenolic compounds were selected for investigation due to their unique structural and mechanistic attributes that distinguish them from other natural product classes. Their multifaceted mechanisms support their potential role as antibiotic adjuvants. Furthermore, extensive evidence supports the translational potential of these natural compounds. These characteristics make phenolic compounds uniquely suited for systematic evaluation in combination therapies, justifying their prioritization over other natural compound classes.

Our findings indicate that synergy between phenolic compounds and antibiotics is not uniform, but rather highly dependent on the mechanistic group of the antibiotic and the chemical class of the natural compound. To determine which structural elements drive this potentiation, we conducted a chemotype-based structure–activity analysis to identify phenolic motifs most strongly associated with synergistic outcomes across antibiotic classes.

Within this heterogeneous class, flavonoids were classified as a distinct chemotype due to their conserved C_6_-C_3_-C_6_ scaffold and associated mechanistic features—including membrane interaction, efflux pump inhibition, metal ion chelation, and virulence modulation—which collectively differentiate them from other phenolic subclasses and justify their independent evaluation in SAR analysis [78]. Overall, flavonoid compounds were strongly enriched for synergy compared to non-flavonoid chemotypes. This aligns with previous studies reporting potentiation of β-lactams by flavonoids through outer membrane destabilization and interference with resistance enzymes such as carbapenemases [79,80,81,82].

The markedly higher synergy observed for meropenem in combination with flavonoids was driven specifically by the presence of catechol and gallate motifs, rather than the flavonoid scaffold alone. These motifs were therefore classified as distinct chemotypes because they confer unique mechanistic advantages beyond the core flavonoid structure. Catechol-containing phenolics possess adjacent hydroxyl groups (–OH in ortho position) that enable iron chelation and reduction (Fe^3+^ to Fe^2+^), driving ROS generation and disrupting metal homeostasis. In MDR *K. pneumoniae*, catechol-containing flavonoids (such as luteolin and myricetin) were shown to potentiate colistin’s efficacy, whereas structurally related flavonoids lacking adjacent hydroxyls showed no synergy [78]. Catechol-mediated iron reduction dysregulates the PmrA/PmrB signaling pathway, increases outer membrane negative charge, and enhances antibiotic binding [83]. These combined effects mechanistically explain the consistent association between catechol motifs and synergistic antibiotic interactions. Additionally, the gallate group (3,4,5-trihydroxybenzoyl moiety) further enhances phenolic antimicrobial potency by increasing lipophilicity and membrane affinity. Galloylated catechins such as epigallocatechin gallate more effectively insert into bacterial membranes, disrupting lipid organization, increasing permeability, and facilitating antibiotic uptake [84]. Gallate substitution also enhances hydrogen bonding interactions with cell envelope components, leading to membrane leakage and interference with cell wall synthesis [85]. Consequently, galloylated phenolics show superior synergy profiles compared to their ungalloylated analogs, supporting inclusion of this motif as a key predictor of antibiotic potentiation.

Overall, flavonoid compounds were strongly enriched for synergy compared to non-flavonoid chemotypes, with catechol/gallol-bearing scaffolds emerging as the most consistent predictors of enhanced activity across combinations.

In contrast, curcuminoid/Michael acceptor and simple phenol classes tended to display lower synergy rates and higher FICI values, indicating less favorable interactions.

Curcuminoids constitute a structurally distinct class of phenolics defined by a linear diarylheptanoid backbone containing conjugated α,β-unsaturated carbonyl groups (Michael acceptors). This electrophilic scaffold enables interaction with nucleophilic targets, modulation of redox pathways, and insertion into bacterial membranes, all of which can influence antimicrobial responses [86]. However, these same electrophilic properties may activate bacterial stress defense mechanisms or neutralize antibiotic-generated ROS, leading to inconsistent outcomes when combined with antibiotics [28,87,88]. In our analysis, curcumin exhibited variable or antagonistic interactions, reflected by higher FICI values compared to catechol- or gallate-bearing compounds. Thus, while curcuminoids possess intrinsic antimicrobial potential, their promiscuous reactivity and redox-modulating effects make them less reliable as antibiotic adjuvants, justifying their classification as a separate chemotype with limited translational potential compared to synergy-promoting motifs.

Simple phenols such as carvacrol were largely neutral, supporting the idea that small hydrophobic compounds exert a more limited adjuvant effect in this context [89].

In our analysis, stilbenes (represented herein by resveratrol) were categorized as a distinct chemotype based on their C_6_-C_2_-C_6_ scaffold, which promotes membrane intercalation, QS disruption, and potentiation of polymyxin activity through unique physicochemical interactions that are not exhibited by other phenolic subclasses. This chemotype was found to possess a very high and consistent rate of synergy with polymyxin B. This is noteworthy, given the increasing clinical reliance on polymyxins as last-line therapies, and suggests that certain stilbene structures may complement their membrane-targeting activity [28,90,91,92]. The consistency of this effect across multiple strains highlights its potential for translation to in vivo models. However, for other antibiotic groups, such as cephalosporins and fluoroquinolones, the evidence base remains limited due to smaller sample sizes.

Glycosylated phenolics such as forsythin and forsythoside B were classified as a distinct chemotype due to the presence of sugar moieties that markedly alter physicochemical behavior, reducing membrane permeability, increasing hydrophilicity, and requiring metabolic activation. These can diminish antimicrobial potentiation compared with their aglycone counterparts [93]. In our dataset, both glycosides yielded an FICI of approximately 1 (no interaction), suggesting that glycosylation may impair their ability to synergize with antibiotics. However, as only single determinations were available for each, these findings should be considered preliminary and hypothesis-generating rather than definitive.

The novelty of this work was the incorporation of FICI variability into the assessment of clinical relevance. While median FICI values provide a measure of central tendency, they can mask high dispersion and strain-specific differences. Our analysis revealed that combinations such as meropenem + quercetin, while often synergistic, displayed considerable variability across isolates, whereas polymyxin B + resveratrol demonstrated both low median FICI and minimal dispersion, making it a stronger candidate for predictable clinical performance. This emphasizes the need to consider reproducibility and stability of effects alongside potency when prioritizing combinations for further development.

The broader implications of our findings are twofold. First, they provide a rational framework for prioritizing natural compounds as antibiotic adjuvants, emphasizing those with structural features predictive of consistent synergy [94]. This is particularly relevant for carbapenem-resistant *K. pneumoniae*, where treatment options are severely limited [95]. Second, the study highlights the value of integrating quantitative synergy metrics with SAR analysis to generate mechanistically coherent hypotheses for future drug development. Moving forward, research should focus on confirming these in vitro findings in animal infection models, elucidating the molecular mechanisms underlying synergy, and exploring pharmacokinetic and pharmacodynamic considerations [96,97]. Additionally, expanding the dataset to include a broader range of phenolic compounds and antibiotic classes will improve statistical power and generalizability.

In summary, this work demonstrates that the potentiation of antibiotics by natural phenolic compounds is structurally determined and highly context-dependent. Flavonoids, particularly those with catechol/gallol motifs, emerge as the most promising partners for restoring carbapenem activity, while Michael acceptor chemotypes warrant caution due to their inconsistent effects. These results lay the groundwork for developing targeted combination therapies that leverage natural products to address the pressing challenge of MDR *K. pneumoniae* and other Gram-negative pathogens.

To translate these promising in vitro interactions into clinical applications, however, it is essential to address the pharmacological limitations of phenolic compounds. Despite their biological potency, many phenolics exhibit poor solubility, limited oral bioavailability, extensive metabolism into inactive forms, and unpredictable pharmacokinetic/pharmacodynamic profiles [98,99,100,101,102,103,104,105]. Overcoming these challenges will require advanced formulation strategies such as nanocarriers, liposomal formulations, and inclusion complexes, which can be employed to improve tissue penetration, ensure targeted delivery, and maintain sustained therapeutic drug levels [106,107,108,109,110]. Additional pharmaceutical solutions—such as co-crystallization, amorphous solid dispersions, nanocapsules, and matrix-based systems—further address bioavailability challenges [111,112,113,114]. The appeal of phenolic compounds is also supported by favorable safety profiles derived from toxicological data, traditional medicinal use, and their marketing as dietary supplements. Nonetheless, issues persist around standardization, dose uniformity, long-term safety, and potential effects on the gut microbiota [115,116,117,118]. Importantly, the ability of low-dose phenolics to restore antibiotic susceptibility and induce synergy supports their cautious advancement in translational research [21]. Still, applying in vitro synergy findings to clinical contexts remains challenging due to pathogen heterogeneity, complex resistance mechanisms, and host-dependent pharmacokinetics [119].

This study has several limitations that should be considered when interpreting the findings. First, the pooled data were derived from in vitro studies conducted under heterogeneous experimental conditions, including variation in media composition, inoculum size, antibiotic concentration ranges, and methodological approaches to FICI determination. Such variability may influence synergy estimates and limit direct comparability across studies, although the stratification by chemotype and antibiotic class was designed to partially mitigate this effect [120].

A potential limitation of our chemotype-based analysis is that individual phenolic compounds often possess multiple structural motifs (e.g., flavonoids simultaneously containing catechol or gallate groups), which may lead to overlapping classification. This structural overlap could contribute to interaction effects that inflate the observed odds ratios for synergy when motifs co-occur, making it difficult to attribute potentiation to a single chemotype. To mitigate this, we stratified compounds using a binary flagging approach and interpreted results in terms of mechanistic enrichment rather than exclusive causality. However, the synergistic effects observed for catechol- and gallate-bearing flavonoids likely reflect combined contributions of both the core flavonoid scaffold and the redox-active functional groups. Therefore, chemotype-specific odds ratios should be interpreted as indicators of association rather than isolated causative determinants, and future multivariate or machine learning models will be required to deconvolute these overlapping effects.

The lack of in vivo validation or pharmacodynamic modeling limits the translational relevance of the observed interactions, particularly given the known pharmacokinetic constraints of phenolic compounds mentioned above.

Finally, the potential for publication bias cannot be excluded. Studies reporting synergistic outcomes are more likely to be published, while neutral or antagonistic findings may be underrepresented in the literature. Although our analysis included combinations across a range of outcomes, including antagonism and no interaction, the reported rates of synergy may overestimate the true clinical potential [121].

Despite these limitations, the study provides a robust framework for identifying structural predictors of antibiotic potentiation and prioritizing phenolic compounds for further investigation in translational models.

## 5. Conclusions

This pooled analysis of 216 in vitro assays shows that synergy between phenolic compounds and antibiotics against multidrug-resistant *Klebsiella pneumoniae* is structure- and context-dependent. The most reproducible effect was observed for polymyxin B + resveratrol, which showed a low median FICI and minimal dispersion. For carbapenems, meropenem + catechol/gallol-bearing flavonoids (e.g., quercetin, kaempferol) outperformed non-flavonoids, whereas curcumin displayed inconsistent or antagonistic interactions. Because synergy magnitude alone can be misleading, low dispersion should guide prioritization for in vivo testing. Future work should validate top low-dispersion pairs in animal models and assess PK/PD and safety to enable clinical translation.

## Figures and Tables

**Table 1:** Comparative in vitro synergistic effects of natural phenolic compounds and antibiotics against multidrug-resistant *Klebsiella pneumoniae*.

Antibiotic	Natural Compound	Reference	n	Median FICI (CI 95%)	Min FICI	Max FICI
Cefotaxime	Baicalein	[60]	16	0.82 (0.69–1.00)	0.38	1.5
Cefotaxime	Cinnamaldehyde	[61]	32	0.455 (0.34–0.58)	0.076	1.5
Cefotaxime	Matrine	[60]	16	1.07 (1.01–1.21)	1.008	2.008
Ciprofloxacin	Cinnamaldehyde	[61]	33	2.15 (1.26–3.31)	0.122	16.06
Ciprofloxacin	Myricetin	[65]	3	1.36 (0.86–2.00)	0.74	2
Colistin	6-Gingerol	[66]	3	0.193 (0.15–0.25)	0.15	0.25
Colistin	Eugenol	[67]	6	0.266 (0.21–0.30)	0.31	0.156
Colistin	Kaempferol	[68]	6	0.27 (0.07–0.50)	0.012	0.75
Imipenem	Epigallocatechin-3-gallate	[69]	26	0.543 (0.44–0.65)	0.15625	1.03125
Levofloxacin	Chlorogenic acid	[70]	3	0.22 (0.16–0.25)	0.16	0.25
Meropenem	Baicalein	[72]	1	0.070 *	0.07	0.07
Meropenem	Carvacrol	[62]	25	0.695 (0.63–0.76)	0.5	1
Meropenem	Curcumin	[63]	39	1.436 (1.05–1.83)	0.001	4
Meropenem	Quercetin	[71]	30	0.5 (0.27–0.86)	0.03	5
Polymyxin B	Resveratrol	[64]	26	0.199 (0.14–0.27)	0.002	0.502
Polymyxin E	Forsythin	[72]	1	1.010 *	1.01	1.01
Tetracycline	Epigallocatechin gallate	[65]	3	0.82 (0.49–1.32)	0.49	1.32
Tigecycline	Forsythoside B	[72]	1	1.01	1.01	1.01

CI, confidence interval; FICI, fractional inhibitory concentration index; n, number of tests. * CI could not be calculated.

**Table 2:** Synergistic activity of natural phenolic compounds combined with antibiotics against multidrug-resistant *Klebsiella pneumoniae*.

Antibiotic	Compound	Reference	n	Median FICI (CI 95%)	KW *p*-Value			Dunn–Bonferroni CLD
					Chi^2^	df	*p*	
Cefotaxime	Baicalein	[60]	16	0.82 (0.69–1.00)	31.77	2	<0.001	A
	Matrine	[60]	16	1.07(1.01–1.21)				A
	Cinnamaldehyde	[61]	32	0.455(0.34–0.58)				B
Meropenem	Curcumin	[63]	39	1.436 (1.05–1.83)	16.83	2	<0.001	A
	Quercetin	[71]	30	0.5 (0.27–0.86)				B
	Carvacrol	[62]	25	0.695 (0.63–0.76)				A
Colistin	Kaempferol	[68]	6	0.27(0.07–0.50)	1.74	2	0.419	NS
	6-Gingerol	[66]	3	0.193(0.15–0.25)				NS
	Eugenol	[67]	6	0.266(0.21–0.30)				NS

Median fractional inhibitory concentration index (FICI) values for each antibiotic-compound combination, limited to those with favorable (≤1.0) median FICI values and sufficient replicates (n) for statistical analysis. Global significance was assessed using the Kruskal-Wallis (KW) test. CI = confidence interval. CLD = *Compact Letter Display*, used for post hoc multiple comparisons (Dunn-Bonferroni). Groups sharing the same letter (e.g., A) are not significantly different, while groups with different letters (e.g., A vs. B) are significantly different. *NS* = not significant.

**Table 3:** FICI summary analysis.

Combination	Reference	n	Min FICI	Max FICI	Range	IQR	SD
Cefotaxime + Baicalein	[60]	16	0.38	1.5	1.12	0.28	0.31
Cefotaxime + Matrine	[60]	16	1.008	2.008	1	0	0.24
Cefotaxime + Cinnamaldehyde	[61]	32	0.076	1.5	1.424	0.314	0.33
Meropenem + Curcumin	[63]	39	0.009	4	3.991	1.84	1.266
Meropenem + Quercetin	[71]	30	0.03	5	4.97	0.32	0.900
Meropenem + Carvacrol	[62]	25	0.5	1	0.5	0.25	0.173
Colistin + Eugenol	[67]	6	0.156	0.312	0.156	0.06	0.062
Colistin + Kaempferol	[68]	6	0.012	0.75	0.738	0.39	0.30
Colistin + 6-Gingerol	[66]	3	0.15	0.25	0.1	0.05	0.051

Legend: FICI, fractional inhibitory concentration index; IQR, interquartile range; n, number of tests; SD, standard deviation.

**Table 4:** Binary structural classification of natural compounds by chemical class and functional motifs.

Compound	Flavonoid	Stilbene	Phenol Simple	Curcuminoid	Glycoside	Alkaloid	Gallocatechin	Michael Acceptor
Baicalein	TRUE	FALSE	FALSE	FALSE	FALSE	FALSE	FALSE	FALSE
Carvacrol	FALSE	FALSE	TRUE	FALSE	FALSE	FALSE	FALSE	FALSE
Curcumin	FALSE	FALSE	FALSE	TRUE	FALSE	FALSE	FALSE	TRUE
Epigallocatechin gallate	TRUE	FALSE	FALSE	FALSE	FALSE	FALSE	TRUE	FALSE
Forsythin	FALSE	FALSE	FALSE	FALSE	TRUE	FALSE	FALSE	FALSE
Forsythoside B	FALSE	FALSE	FALSE	FALSE	TRUE	FALSE	FALSE	FALSE
Kaempferol	TRUE	FALSE	FALSE	FALSE	FALSE	FALSE	FALSE	FALSE
Matrine	FALSE	FALSE	FALSE	FALSE	FALSE	TRUE	FALSE	FALSE
Myricetin	TRUE	FALSE	FALSE	FALSE	FALSE	FALSE	TRUE	FALSE
Quercetin	TRUE	FALSE	FALSE	FALSE	FALSE	FALSE	TRUE	FALSE
Resveratrol	FALSE	TRUE	FALSE	FALSE	FALSE	FALSE	FALSE	FALSE

**Table 5:** Summary of synergy (FICI ≤ 0.5) and antagonism (FICI ≥ 4.0) by structural class.

Structural Feature/Group	% Synergy (95% CI) Flag Present	% Synergy (95% CI) Flag Absent	OR (Synergy)	RD	*p* (Fisher)	Δ-Median log_2_(FICI) (HL Estimate)	*p* (Mann–Whitney)	Approx. Fold Change in FICI
Flavonoids	64.9 (51.9–76.0)	23.8 (15.8–34.1)	5.94	41.2	1.6 × 10^−6^	−1.415	3.7 × 10^−7^	↓ 2.7
Catechol/Gallol motif	80.9 (66.7–90.0)	23.2 (15.8–32.6)	14.1	57.8	2.5 × 10^−10^	−1.95	1.3 × 10^−8^	↓ 4
Curcuminoid/Michael acceptor	30.8 (18.6–46.4)	44.9 (35.4–54.8)	0.55	−14.1	0.18	0.99	0.016	↑ 2
Simple phenols (carvacrol, eugenol)	28	56.8	0.3	−28.8	0.02	1	0.12	↑ 2
Phenolic acid	100 (43.9–100)	51.1	–	–	0.248	–	–	–
Alkaloid	0	54.9	~0.02	−51.9	5.38 × 10^−6^	–	–	–

Legend: CI, confidence interval; FICI, fractional inhibitory concentration index; OR, odds ratio; RD, risk difference; ↓ decrease; ↑ increase.

## Data Availability

The original contributions presented in this study are included in the article. Further inquiries can be directed to the corresponding authors.
